# Medical Tourism in Iran, Reevaluation on the New Trends: A Narrative Review

**Published:** 2019-07

**Authors:** Ladan ROKNI, Sam-Hun PARK

**Affiliations:** Asia Contents Institute, Konkuk University, Seoul, South Korea

**Keywords:** Medical tourism, Medical travel, Health tourism, Global trends, Iran

## Abstract

**Background:**

This narrative review aimed to reevaluate the medical tourism sector in Iran to reveal the new trends and activities in order to know the current actual share of Iran in the competitive market of global medical tourism.

**Methods:**

A holistic approach was adopted to analyze the information collected through a system of investigation that comprised the available secondary data, besides the information and statistical data about the associated organizations to this sector in Iran.

**Results:**

Iran’s medical tourism sector is consistent with the new trends in global market, which is mainly bordered countries-based, cultural-oriented, and diasporic type of travelling.

**Conclusion:**

It would be more realistic in the contemporary situation to focus the promotions on the border countries, and design the policy, and implementation in accordance with their cultural and social preferences. Activities in both levels of governmental and micro-level planning are demanded, under the cover of a comprehensive monitoring system. This review will benefit researchers exploring the updated evaluation of Iran’s medical tourism; also, it provides helpful insights to authorities in both governmental and private sector.

## Introduction

Investigating the main trends and mechanism of the medical tourism sector, have been lately announced globally as a necessity due to the fast-growing changes and diverse information from around the world. The data for medical tourism are revolving globally.

Lately, the pioneers of medical tourism reported that this sector is ‘relatively short distance, cross border and diasporic’ (1). In the latest report by ‘International Medical Travel Journal’ (IMTJ) (2), also, the new transformations in this sector are announced. Based on this report in 2018, regional or domestic medical travel makes the greater proportion, and medical tourists are no longer seeking out the cheapest destination (2). However, the ‘ease and affordability of international travel’ are still among the pull factors for medical travelers besides the improvements in technology and standards of care. In this report, the failure of several medical tourism projects makes this alarm for the authorities who are tending to develop medical tourism in their organizations/countries. It is estimated that the annual number of medical travelers worldwide is 5.5 million and the value of medical travel expenditure is US$10–15 billion (2). Medical tourism is estimated to be an industry with a value of $100 billion dollars in future (3).

Despite the considerable development and industry growth, we know very little about the mechanism of medical tourism in Iran. Most of the available researches are based on the previous knowledge of medical tourism, in spite of the contemporary revolving system. Iran has been popular for doing the treatment during the travel (4), especially family travel from Gulf countries to Shiraz and Mashhad, two cities in Iran (5). However, Based on the MTI (medical tourism index), Iran is not among the 30 destination countries evaluated in 2016 (3). Among the very diverse information and figures, once, Iran has been reported as the 10th top country in terms of the value in medical tourism, by IMTJ (Table 1).

**Table 1:** Top ten medical tourism destinations, based on value and numbers

***Top ten medical tourism Destinations by value,***	***US $m***	***Top ten medical tourism Destinations by numbers in, 000K***	
USA	3,500	Malaysia	900
South Korea	655	USA	500
Turkey	600	South Korea	365
Thailand	600	Thailand	350
Germany	575	Dubai	350
India	450	South Africa	300
UK	350	Taiwan	300
Malaysia	350	Germany	255
Mexico	350	Mexico	250
Iran	315	Turkey	200

Source: Medical travel and tourism market briefing, 2018 (2)

Iran was considered the Middle East’s top tourist destination during the period 1967–1977. The political changes in the following years affected the international inbound tourism to Iran, and the balance has shifted away from Western sources to -mostly- religious tourism from other Muslim countries (6). Table 2 comprises the amount of capital investment in tourism by three countries in the region, which represents the investment by Iran as, relatively insignificant. There are two major challenges, which also affect the medical tourism sector: ‘Iran’s negative image in the West, and lack of resources to tackle this negative discourse’ (7).

**Table 2:** Capital investment in tourism Iran vs. UAE/Turkey Source: World Travel and Tourism Council (2014)

***Capital investment US$***	***2005***	***2006***	***2007***	***2008***	***2009***	***2010***	***2011***	***2012***	***2013***
Iran	1.86	2.3	2.49	3.28	3.22	3.233	3.773	3.803	4.166
UAE	3.67	5.76	13.3	16.9	14.2	17.93	20.821	22.541	24.848
Turkey	9.39	9.41	8.32	11.3	11.9	10.32	17.326	16.941	18.523

Several competitive advantages, meanwhile, are listed for Iran’s medical tourism, ranging from “the valuable medical resources, infrastructure and medical skills” to “cultural and social similarities with the border countries”, besides “religion-based factors”, and also a considerable number of Iranian living in foreign counties, who prefer to travel back to their country of origin for treatments (diasporic tourist) (8). It is projected that by the perspective of the year 1400 (2021), 550000 foreign patients will visit Iran, and it will lead to 2750000 dollar income and creating 392857 direct and indirect job opportunities (9).

However, all these projections for the future are ‘unrealistic expectations’ (10) since there are several barriers to reach the claimed points. More important though, are some missed factors that can potentially lead to the development and promotion in more realistic pathaway. Investigation regarding the actual information and mechanism has been limited and narrow in scope, and based on the former beliefs in medical tourism arena. Offering new policy and planning in accordance with the new changes would not be possible and effective, unless a comprehensive inquiry is provided.

Accordingly, this study aimed to investigate the contemporary situation of medical tourism sector in Iran in order to provide new insights based on the updated medical tourism system in the world. To be more, specific, the objective was to investigate the situation based on the secondary data and the available statistical information, with the aim of evaluating this sector in accordance with the new changes. The implication of this research can assist managers in both governmental and private sector in Iran to arrange their policy and strategies based on the global updates in medical tourism sector.

## Methods

This study is a narrative review based on the available secondary data, besides the information and statistical data about the medical tourism sector in Iran. A holistic approach was taken into account for the analysis of Iran’s contemporary situation as a medical tourism destination.

In November 2018, we searched the well-known academic publication databases. We also manually searched the bibliographies of key articles in well-known journals. Table 3, represents the information and contents of the published scholarly articles about the medical tourism in Iran, so far. We collected and studied every single article, and rewrote the main concepts of each, and then the articles were given different categories based on the type of research.

**Table 3:** Published scholarly articles on medical tourism in Iran

***Reference No***	***Aim & method***	***Findings***	***Type of research***
(11)	Searching the pull factors of Mashhad to attract MT	Factors from the perspective of patients: staff, service and process gained the highest score, while price, facilities and promotion scored the lowest	Demand side
(12)	Evaluating MT in Yazd province through a developmental approach	A strategic planning framework was introduced for development of medical tourism industry in Yazd	Case study
(13)	Examined the effect of trust and religiosity on Islamic medical tourists’ attitudes	Although trust has a significant effect on Muslim medical tourists’ attitudes, religiosity has no significant effect. Gender and education moderate the relationship between religiosity and attitude	Demand side
(14)	To determine factors impeding the development of medical tourism in East Azerbaijan	Barriers: Marketing, international issues, culture, transfer, brokerage, management, and policy	Case Study
(15)	To evaluate the situation of west Azarbaijan province	Provided local strategies for development	Case Study
(16)	To explore the required information to design a MT website	Several required themes were introduced	Marketing
(17)	Inquiry on the Tourists’ Satisfaction in Lorestan	Problems: personnel, equipment, and medical and welfare facilities’ shortage, skillful personnel, mismanagement	Demand side
(18)	Investigating the importance of training in MT in Iran	Not only equipment, but also the necessary training should be provided.	General
(19)	Identifying the barriers to develop MT in Iran	General results	General
(20)	Searching on the effective factors on development of health tourism	A meaningful relationship between the province economic and social infrastructures as well as macro planning and management and its health tourism development.	Case study
(21)	To search the effective factors on expansion of MT in Iran	“Healthcare quality” and “high level of expertise” are two most attractive factors, besides, costs, and visa facilities // The role of “the healthcare providers” is more prominent	General
(22)	To identify and prioritize the effective factors in attracting health tourists in Tehran	Quality, patient-centered areas, and appropriate time were ranked first to third	
(23)	To examine the impact of medical travelers’ behavioral intention in Iran	Positive relationship between destination image, service quality and patient satisfaction, attitude, and revisit intention	Demand side
(24)	Survey the factors in development of health tourism in Iran	Health tourism branding, correlation between different sections, and centralized services are 3 main factors in development of health tourism of Iran	General
(25)	Searching for the barriers on the way of developing, edical tourism in Iran as a national goal	Provided the general obstacles and associated suggestions	General
(5)	To evaluate the situation of Shiraz City in terms of MT	Cultural and religios familiarity, family relationships, besides the level of trust to Iranian physicians have made Shiraz as a known destination in the region	Case study
(26)	Searching for the marketing-based developmental model in Shiraz	Hospitals were in the best condition regarding staff and physician, and in the worst condition concerning promoting and facilities	Case Study
(27)	Asking medical tourists perception	The poor English language skills will impact the communication	Demand side
(9)	Searching the opportunity or threat of health tourism in Iran	Strengths: expert physicians, up-to-date medical technology weaknesses: poor coordination among the organizations, and planning.	General
(28)	Searching for the positive and negative sides of MT of Iran	General results	General
(4)	Investigating the decisive factors in destination choice, focusing of the reproductive treatment	Religious affinity is important in reproductive medical tourism for Muslim infertile couples	case study
(8)	Analyzing the challenges and opportunities of MT in Iran	The competencies of Iran’s MT should be applied for development and to attract the neighbor countries	General

The available information on the website and country profile of IMTJ was totally studied, because this database aims to create a central focus for information, resources and opinion on medical travel, and has a profile of specific information for each country; also, it presents discussion and articles for each country.

Besides, the Google search engine was used to obtain narratives, news, blog posts, links and associated organizations’ websites on medical tourism in Iran, both in English and Farsi.

Eventually, we tried to classify the collected information and present a logical explanation and interpretation of the contemporary situation, both in the research arena and in reality.

## Results

The results of the investigations are presented in two different sections: published articles, and collected information. The first section provides a comprehensive view and deduction on the published articles and represents the actual contemporary situation of how medical tourism has been evaluated by scholars.

The second section comprises several subtitles that provide the updated information and the associated interpretations.

### Published Articles

Even though most of the articles have tended to investigate the opportunities and challenges of medical tourism sector in Iran, there are very few articles published in well-known international journals, and we are facing lack of the research that presents reliable data collection and analysis on the contemporary situation, both qualitative and quantitative.

However, there are number of researches that present very helpful insights for authorities and could, so far add to the literature. Nevertheless, the main general output of these articles are as follows:

- A comprehensive managerial solution is required in Iran’s medical tourism section, in which the strategic plan can be suggested for development;
- There are several organizations active in medical tourism, and what is highly required is to design a system of coordination between these sections;
- In order to control the illegal activities of some people and organizations (especially the brokers), designing a centralized service can potentially address many shortages;
- Even though a centralized managerial system (policy) has been mentioned, the importance of having macro planning was not forgiven by researchers. It is critical since it will lead to development;
- In many pieces of research, local strategies are provided based on the province, which seems helpful in some cases and can assist the managers, however in some articles solutions are presented in a very general form and are somehow duplication of the familiar challenges in the previous publications;
- The image of Iran is not acceptable enough in the mass media to convince tourists to visit Iran, and it might be because of lack of the appropriate contents in the hospitals’ websites, and researchers called for a systematic promotional plan;
- Training and having skillful healthcare practitioners was a matter mentioned in many pieces of research. It ranged from English professions or Arabic in some cases, to interaction competencies;
- The role of healthcare providers was considered more powerful than the other services. The direct interaction of doctor-patients makes it very critical and effective in terms of the satisfaction and eventually the success of the whole service.

### Collected Information

#### Medical Tourism in Iran

The IMTJ, reports annual statistics and information for each country that offers medical tourism. We mainly based our search on their information, as a globally reliable database. The other information is collected from governmental and private websites in Persian.

Iran with the majority of Shi’a sect population, is located in the Middle East, and is surrounded by Muslim countries. Many cities of this country have been historically famous as a tourist destination in the region, specifically, Shiraz, Isfahan, Yazd and Mashhad. To be more specific, the cultural and social similarities, besides the familiar family backgrounds caused the border countries citizens to choice Iran as their destination (5). The popularity of Iranian health practitioners in the region and the qualified medical facilities has been another main reason for Iran to be considered as a medical tourism destination (5, 9); Religion, also, has been always an effective factor to attract tourists and medical tourists from the border countries; in terms of fertility treatment, which has different rules in Shi’a and Sunni branches (29), and in a practical research in Isfahan, 94% of the participants (foreign patients) were Shi’a Muslim and other 6% were Sunni Muslim, with the country of origin of, Iraq, Afghanistan, Pakistan, in order (4).

Iran also offers several other comparative advantages, including low-cost healthcare system, successful performance of surgical procedures (30) (especially transplantation of liver, heart, eye treatments, fertility), and up-to-date medical technology and natural healing regions (9).

#### Managerial Structure

Both private and public sectors are active in medical tourism sector in Iran. There is a ‘Health Tourism Council’, under the covering of the Ministry of Health, formed in 2015, which comprises representatives from the Ministry of Foreign Affairs, Ministry of Health, Iranian Cultural Heritage Handicrafts and Tourism Organization (ICHHTO), and Iran Medical Council. The primary task of this council is to ‘review and approve regulations that directly affect hospitals catering to foreign patients, and also the travel agencies’ (31). In addition, it is their authority to evaluate and issue the IPD (International Patient Department) certificate for hospitals, in which hospitals are allowed to admit foreign patients. The Ministry of Health has been in charge of organizing education programs. In 2016, the first steps in training people and companies were taken into account. This ministry also has developed 6 mandatory guidelines for those medical centers that aim to provide services for foreign patients, including ‘the general condition of the facility, the workforce, medical facilities, geographical location, operational conditions, and the content of websites’. They further offer facilities to hospitals, such as marketing and training (32).

Lately, well-structured and private organizations started working and offering different services as authorized brokers and service providers. Such services caused development in the sector since they could fulfil the requirements of foreign patients specifically in accordance with their cultural and social background and their linguistic preferences.

#### Fact and figures

Unfortunately, the annual statistics regarding the number of medical tourists entering Iran are unavailable. There are several reasons, that we classified them in 4 categories: 1) Due to the lack of a systematic recording system for the foreign patients in country; 2) Even though, some statistics are available, yet there are many unauthorized organizations and persons that are not counted in the statistical data; 3) In some cases ‘diasporic tourists’ (traveling their country of origin for treatment, and a remarkable number of Iranian are living in foreign countries) also are counted in this information, which make the data untrustworthy; 4) Also another problem is the confusion between the meaning of medical tourism and health tourism, the figure also include those people going to hot and cold springs (33).

**The number of foreign patients:** Accurate statistics are accessible from 2004 and 2005, in those years 12,000 and 17,500 patients were treated, respectively (14). Iran had nearly 30.00 foreign patients in 2012, and this has gone up to almost 200.000 in 2015 (34). In 2016, Iran had 105,000 health and medical tourists (10). According to Iran’s Medical Tourism Department, official statistics confirm over 300.000 foreign patients during the 2017–2018 (Iranian calendar year 1397–1398). According to Iran document 1404 (2025), it is predicted that 1,400,000 people will be attracted to medical tourism (14). In addition, it is projected to attract around 550.000 medical tourists every year (35), but this is also unrealistic due to the several reasons, mentioned in the following sections.

**Country of origin and the destination cities:** Foreign patients generally came from Iraq and Afghanistan, and the main destinations were Mashhad and Tehran (35). In accordance with Iran’s governmental report, Afghanistan, Iraq, Pakistan, Azerbaijan and Oman sent the most number of patients to Iran in 2017 (36).

**The number of active organizations:** Regarding the number of the authorized active organizations, in 2016 the Ministry of Health has authorized 98 hospitals to admit international patients (IPD). In 2017, the numbers of hospitals have increased, taking the total to 170.

**Economic revenue:** Iran’s annual revenue from medical tourism is between $400 and $500 million, announced by ICHHTO, while the target is to reach $2.5 billion. Each person spends between $3.600 and $500 million every trip (33).

The Ministry of Health believes that Iran has the capacity to earn $7 billion in medical and health tourism, though the real data is less than $1 billion (37). However, the target is unrealistic and the figure is not going to happen claimed by a member of Iranian Ministry of Health (10).

#### Push and Pull Factors on Iran’s Medical Tourism Sector

**Fig. 1:**
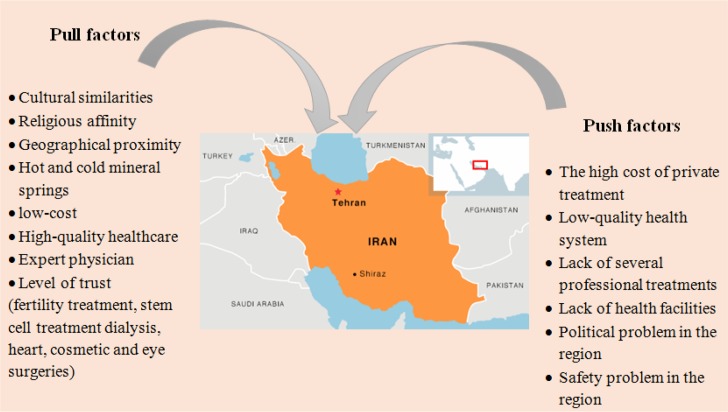
Opportunities in Iran’s medical tourism. Designed by authors, information collected from the secondary data

Fig. 1 was designed based on the collected secondary data, articles presented in Table 1, and the interpretations by IMTJ. Opportunities in Iran’s medical tourism are presented based on the push factors in nearby countries and the pull factors available and offered in Iran.

In addition to some general factors, which motivate all groups of international medical tourists (such as price, distance, lack of expertise, and tourist attractions), there are additional important factors in Iran’s medical tourism sector. Offering ‘medical service based on the religious belief’ (cultural and social familiarity) is one main factor, and another factor, which specifically influences infertile couples’ destination choices is the ‘qualified infertility treatment’ which is offered in Iran, unlike the border countries (4).

#### Associated Problems to Iran’s Medical Tourism

As noted in Table 3, many researchers have tended to clarify the associated barriers to the development of medical tourism. Here, we tried to classify the shortages and problems in the following categories:

**The activity of the middleman (brokers):** Their role is critical and vital for medical tourism in Iran since the sector was started in the country through their activities in promoting trust. However, these days the unlicensed brokers are believed to be Iran’s biggest issue in medical tourism (38). There is a need for ‘eliminating unauthorized middleman’ as it is believed that they create a negative image for Iran (39) rather promotion. This problem can be solved through monitoring and the harmonization of the prices and services, and it can prevent the potential upcoming risks for both the patients and the image of the country.

**Comprehensive policy and monitoring system:** Even though, the managerial system has been mentioned several times as a shortage, it should be noted that the problem is not just to set and review the regulation. In the contemporary situation, there is a need for an organized monitoring system on the activity.

For instance, many hospitals are offering the government approved tariffs for foreign patients, however, some hospitals or doctors, individually, charge their patients with some high prices, especially after the inflation and exchange rate problems in the country. Such problems cannot be solved unless a powerful monitoring system is designed.

Different sectors are active for medical tourism in Iran. It is believed that private sector should lead the industry, offered by Iran’s health minister (40), and the competition should be in terms of the service quality and cheaper prices. Nevertheless, the alliance is highly required among all the active sectors, and at the same time, the actions and strategies can be practiced in a micro level manner, which is in accordance with a national and comprehensive viewpoint.

**Education:** The Ministry of Health lately offers specific training workshops for individuals active in medical tourism. These lectures are newly established and surely need improvement during the time, meanwhile, the main problem is because it is offered (mainly) from the clinical perspective and non-clinical perspective still need to be considered in the educational system. Medical tourism demands for skilled personnel in both medical and welfare services. Language skills also are critical for appropriate interaction, which needs an inclusive education for general improvement.

Iran is a country full of potential for being among the main destinations for medical tourism, at least for the regional countries, however, still, there is a way to reach to that point since both brokers’ organizations and hospitals need specific training (https://medtourpress.ir/).

**Political perspective**: Iran’s tourism industry has suffered significantly over the past decades because of a number of issues, which all have led to a negative destination image of this country and the situation (41–43). The political stability can give Iran the time to revive and develop its tourism industry, which can create a better understanding of this ‘misinterpreted nation’ (6).

It is inevitable also for the medical tourism sector to remain untouched by these political factors. Even though the border countries are familiar with the actual situation and they are well informed about the safety of travelling Iran, still there are some legal challenges for agencies active in medical tourism, due to the sanction and they are facing barriers for collaboration with the foreign companies (44).

**Marketing and promoting:** Attracting foreign patient in this competitive market cannot be achieved unless suitable marketing is provided to convince the patients to choose Iran as their treatment destination. Iran now suffers from lack of a well-established media and appropriate contents in the designed websites and brochures for introducing the facilities and infrastructure. In a better word, there is a lack of a comprehensive information management system specific to medical tourists (14, 27).

Non-clinical facilities and services: Iran has been always popular concerning the medical expertise, however, medical tourism demands for a package of service, which comprises both clinical and non-clinical services. This country is still far from the standards and the luxury services provided by the top medical tourism destinations around the world. It includes both the infrastructure, which is insufficient (14) and skilled service providers in accordance with the cultural and social preferences that offer welfare services (17).

## Discussion

Due to the latest changes in the trends of medical tourism sector around the world, this research aimed to reevaluate the data of this sector in Iran to find the main activities and trends, and evaluate the extent to which Iran is in line with the new trends.

Consistent with the newly announced changes (1, 2), the medical tourism sector in Iran is also ‘border countries-based, short distance, cultural-oriented, and diasporic’ type of medical tourism. Iran also has capabilities in terms of the most popular medical sectors for global medical travels, namely cosmetic, fertility or dental treatment (2) since the services are highly advanced and qualified in these three sectors, besides several other offered advantages that are consistent with the new global trends and even the projections. For instance, it has been claimed by an active pioneer in medical tourism branding, that ‘human behavior will dictate the future of medical tourism marketing’ (45). Likewise, this sector in Iran owes to the cultural and social similarities with the border countries (where medical travels are highly demanded), and the level of family-based trust to Iranian doctors’ expertise which is an efficient factor in the promotion of Iran’s medical tourism (5, 32).

Cultural competency of healthcare providers has been always critical in the scope of healthcare services for foreign patients, and it can provide an advantage in this competitive arena of medical tourism (46–49). Iran, due to the religious-oriented factors and cultural familiarity with the border countries, is highly capable of being introduced as a medical tourism destination in the region that offers services in accordance with the cultural and social background and preferences. For instance, it was shown that ‘providing an appropriate environment where infertile couples’ religious beliefs are respected and considered during the treatment of infertility through ART, will make reproductive tourism a remarkable opportunity’ for Iran’s medical tourism (4). Though the role of “the healthcare providers” will always talk first (21, 50) and it could be even more prominent than the roles of other factors, such as “the government” and “the general tourist services” (21). Searching on the most attractive factors, it was found that the staff has the highest effect of attraction of foreign patients, while ‘price’ shown to be the lowest (11), and offering patient-centered care is of high importance (22). Since two factors of ‘education’ and ‘gender’ can affect the attitudes of the foreign patient visiting Iran (13), more evaluation on the biographical characteristics is required which can be addressed through an active and efficient system of information recording.

Although this sector in Iran offers numerous capabilities and advantages, it faces diverse challenges (discussed in the previous section) as well that act as barriers for the promotion, and demands for further developmental remedies. The main and most important problems are the unauthorized brokers, lack of an efficient promotional and marketing system, and lack of an active monitoring system. More participation and investment of the private sector can be a successful remedy in this case (40). Although the image of Iran is not acceptable enough to convince tourists to visit, researchers suggested that this issue could be solved through local strategies and for the neighbor countries (7). Due to the image of the country in western mass media, it would be more realistic to focus the marketing for the border countries and offer the promotional remedies at a macro level. Providing a well-qualified service for patients coming from border countries can potentially change the general attitude and lead to development.

Considering the contemporary situation, the main solution to address the requirements in the country is to design a comprehensive ‘digital platforms’ (38) that offers harmonized services and centralized system, prices, and other associated factors for each provided package separately. Moreover, using a well-established integrated information system can assist the process of monitoring over the medical tourism practices around the country, which is far more demanded than just offering and setting the regulations. Organizations and authorities in both levels of government and private sectors (micro-planning level), are called to design and implement their strategies through very efficient collaboration. Regarding the brokers, also, in spite of the unauthorized person, their activities cannot be deleted totally, since they promote the market, and monitoring will eliminate the associated problems.

Well-structured private organizations and authorized brokers can directly fulfil the cultural, social, and linguistic requirements of foreign patients, and it would be the best solution to promote the marketing mix and paying more attention to the micro-level advertisements (14, 26) and offer the services in accordance with the cultural-oriented factors. In this regard, it also has been suggested to design the country’s macro policy in a way that promotes interaction with border countries (14). Eventually, considering the introduced push and pull factors in the process of planning, management, and implementation will assist the authorities to design the best strategies accordingly.

## Conclusion

The medical tourism sector in Iran, due to the contemporary situation is consistent with the global trends, which are border-based, short distance, cultural-oriented, and diasporic. Accordingly, the most realistic strategies would be the act of focusing on the marketing and promotion for the foreign patients from neighbor countries, and the positive outcome will not occur unless a combination of centralized policy, micro-level planning, and cultural-oriented factors are carefully taken into account.

## Ethical considerations

Ethical issues (Including plagiarism, informed consent, misconduct, data fabrication and/or falsification, double publication and/or submission, redundancy, etc.) have been completely observed by the authors.
